# Long-term outcomes of lower limb post-traumatic osteomyelitis

**DOI:** 10.1007/s00068-022-02104-9

**Published:** 2022-09-17

**Authors:** Paul Rodham, Michalis Panteli, Catherine Qin, Paul Harwood, Peter V. Giannoudis

**Affiliations:** 1 LIMM Section Musculoskeletal Disease, Academic Department of Trauma and Orthopaedics, School of Medicine, University of Leeds, Leeds General Infirmary, Clarendon Wing, Level A, Great George Street, Leeds, LS1 3EX UK; 2 North East Thames Foundation School, Barking, Havering and Redbridge University Hospitals NHS Trust, Romford, UK; 3 Department of Trauma and Orthopaedics, Leeds Teaching Hospitals NHS Trust, Leeds, UK

**Keywords:** Osteomyelitis, Outcomes, Trauma, Limb reconstruction

## Abstract

**Purpose:**

Whilst recurrence and amputation rates in post-traumatic osteomyelitis (PTOM) are described, limb specific functional outcomes are not, leading to a knowledge gap when counselling patients prior to management. We aim to investigate the patient reported outcomes (PROMS) of this patient group to provide reference for discussions with patients prior to embarking on treatment.

**Methods:**

Single institution cross-sectional retrospective study of all patients presenting with PTOM of the tibia/femur over a 7-year period. Alongside recurrence and amputation rates, patient reported outcomes were recorded including the lower extremity functional scale (LEFS), EQ-5D-3L and EQ-VAS.

**Results:**

Seventy-two patients (59 male; median age 46 years) were identified. Treatment was principle-based and included debridement (with Reamer–Irrigator–Aspirator (RIA) in 31/72), local antibiotics (52/72), soft tissue reconstruction (21/72) and systemic antibiotic therapy in all cases. PROMS were collected in 84% of all eligible patients at a median of 112-month post-treatment. Twelve patients experienced recurrence, whilst nine underwent amputation. The median LEFS was 60, the EQ-5D-3L index score was 0.760, and the EQ-VAS was 80. These scores are substantially lower than those seen in the general population (77, 0.856 and 82.2, respectively). LEFS was significantly higher, where RIA was utilised (69.6 vs 52.8; *p* = 0.02), and in those classified as BACH uncomplicated (74.4 vs 58.4; *p* = 0.02). EQ-5D-3L was also higher when RIA was utilised (0.883 vs 0.604; *p* = 0.04), with no difference in EQ-VAS scores.

**Conclusions:**

Patients with PTOM report functional outcomes below that of the general population, even when in remission. Improved outcomes were associated with uncomplicated disease and the use of RIA.

## Introduction

Osteomyelitis is a progressive inflammatory process, caused by infectious agents, that can lead to bone destruction and sequestra formation. In the developed world, it occurs in adults most frequently as a result of trauma and subsequent surgery [1–3]. The risk of osteomyelitis varies with rates of less than 1% reported in closed injuries, and as high as 50% following open fractures [3, 4]. Infection likely occurs following direct inoculation at the time of injury or during surgery, the risk being exacerbated by damage to the local blood supply and soft tissue envelope [5]. In open fractures, increased time from injury to administration of antibiotics and debridement have both been associated with an increased risk of osteomyelitis [3, 6–8]. Other risk factors include diabetes mellitus, hypoxic lung disease, renal or hepatic failure, major-vessel disease, smoking, peripheral neuropathy and increasing age [9–14].

Treatment options for post-traumatic osteomyelitis (PTOM) include a non-operative, supportive approach with management of acute exacerbations, limb reconstruction surgery with the intention of eradicating infection, and amputation. Decision making is based on the patient’s wishes, the severity of their symptoms, the complexity of the pathology, and the patient’s general medical condition. Surgical treatment can be complex and usually includes debridement of all devitalised tissues, dead space management and formal soft tissue cover when required [15, 16]. Where implants are present, these will require removal in the chronic phase with simultaneous management of non-union if the fracture has not healed [17–20].

Management of chronic osteomyelitis will often be protracted, and late recurrence is not uncommon [21, 22]. Complex surgery will potentially cause further damage to an already injured limb, and the level of function achieved even following successful treatment may well be poor. Despite this, few previous studies have specifically examined outcomes in patients following the management of PTOM. Those that exist are limited by small sample sizes and short follow-up. Such information is important in counselling patients who are considering different treatment options. The aim of this study, therefore, is to investigate the long-term outcome of operatively managed tibial/femoral PTOM to determine (1) what are the long-term recurrence rates? (2) what are the long-term amputation rates? and (3) do patient reported outcome measures return to that of the normal population following successful treatment?

## Patients and methods

The study plan was approved by our local institutional review board (Institutional review board number #5689). A cross-sectional retrospective series of patients presenting to our institution between September 2009 and August 2016 was conducted, with patients identified from our prospectively assembled database. Patients aged 18 years or older, with a confirmed diagnosis of PTOM of the tibia or the femur defined according to the 2017 consensus definition for fracture-related infections (FRI) were included [23]. Exclusion criteria comprised osteomyelitis without preceding trauma, osteomyelitis secondary to septic arthritis, osteomyelitis following pathological fractures and patients followed-up at other institutions. Patients undergoing non-operative management or presenting with infected non-unions were not included in this study due to the different treatment strategies, and therefore, outcomes expected in these patient groups.

Information contained in the database was supplemented by a comprehensive review of the patient’s case notes and imaging. Demographics and clinical details including age, gender, injury characteristics, overall injury severity, details of treatment strategy, organisms cultured from deep samples, and duration of orthopaedic follow-up were recorded. Overall injury severity was quantified using the Injury Severity Score [24]. Osteomyelitis was classified according to both the Cierny–Mader and the BACH classifications [25, 26].

Patients were asked to provide patient reported outcome measures (PROMS) having been invited to attend a face-to-face clinic. This included the collection of the Lower Extremity Functional Score (LEFS) [27] and the EuroQol EQ-5D-3L, and the EQ-VAS [28].

Missing data and loss to follow-up, both of which may bias results, are clearly reported. The characteristics of patient groups in these situations were compared to ensure that there were no fundamental differences in demographics or treatment strategy.

### Clinical management

A management strategy was selected based upon the patient’s wishes, guided by the treating clinician. Treatment was led by a consultant orthopaedic surgeon working alongside a consultant plastic surgeon, where the quality of the soft tissue envelope was a concern.

For limb salvage, debridement of the devitalised bone and soft tissue was undertaken. The Reamer–Irrigator–Aspirator (RIA) system was used to augment debridement, where intramedullary sepsis was present. Further operative management included dead space management, delivery of local antibiotics via absorbable or polymethyl methacrylate spacers and soft tissue coverage where required. Antibiotic prophylaxis was administered after deep samples were obtained for microscopy and culture.

Where amputation was selected as treatment, the patients were reviewed and counselled pre-operatively by clinical psychology and amputation rehabilitation services. Level of amputation was determined in consultation with the rehabilitation services based upon limb function and the location of infection with the intention of clearing all infected tissue.

Antimicrobial therapy was prescribed based upon advice from consultant microbiologists. Broad spectrum antibiotics were given post-operatively until culture results were available. Where cultures were negative, empiric antibiotics were continued at the discretion of the treating clinician and microbiology consultant. A plan for 6 weeks of systemic therapy from the definitive debridement was usually made, dependent on the clinical context. Whilst a recent Cochrane review demonstrated that there was no evidence for a specific duration or route of antibiotics, most treatment regimens usually provide antibiotics for 4–6 weeks [29]. Patients were regularly followed up in the orthopaedic clinic until it was determined that their situation was stable, and the treatment appeared to have been successful. Long-term follow-up was then arranged with patients only discharged when their functional recovery had reached a plateau and they remained infection free. All patients were provided with information on how to arrange urgent review from the service if they had symptoms of recurrent infection in the future.

### Statistical analysis

Statistical analysis was performed using Prism 5.0 (GraphPad Software, CA, USA). Assumptions for parametric analysis were not met; therefore, for numeric variables, the central tendency is described as a median and spread by the interquartile (IQR) and absolute range. Non-parametric methods (Mann–Whitney *U*, Kruskal–Wallis, and Spearman’s rank tests) were used to examine relationships between variables. Nominal variables were compared using Chi-squared and Fisher Exact tests as appropriate. Statistical significance was assumed at *p* < 0.05.

## Results

### Patient demographics, classification and treatment

Out of 119 patients that were diagnosed with chronic osteomyelitis, 72 patients met the inclusion including 59 males and 13 females (median age 47 years, IQR 24, range 18–72). PTOM affected the tibia in 46, and the femur in 26; diagnosed at a median of 14 months following injury (IQR 36, range 5–122). Nineteen patients developed infection following an open fracture.

Of the 72 patients treated, 67 patients were initially treated by limb salvage. Fifty-six patients had orthopaedic implants removed (9 had previous implant removal, 2 never had implants in situ), thus no patient had an implant left in situ following this cycle of treatment. In 30 patients, the Reamer–Irrigator–Aspirator (RIA) system was used for debridement of the medullary canal. Forty-seven patients had local antibiotic insertion [antibiotic nail (22), antibiotic beads (5), antibiotic cement (15), or a combination of these techniques (5)]. Twenty patients required a soft tissue reconstruction as part of their operative management, including ten free-flaps, four pedicled flaps, one skin graft, and five, where a combination of these techniques was utilised. Patients underwent a median of 3 procedures (IQR 2, range 1–13) and a clinic-based follow-up of 23 months (IQR 25, range 10–119).

Details of causative organisms identified on deep culture are presented in Table 1. The most commonly isolated organism was *Staphylococcus* affecting 28 patients (42%). All patients undergoing limb salvage received systemic antibiotics during the post-operative period. These were delivered via the IV route only (6% patients, mean duration 42 days), oral route only (38% patients, mean duration 70 days), or IV then oral routes (56% patients, mean duration IV 14 days, mean duration oral 30 days).

**Table 1:** Organisms isolated from PTOM cohort (*n* = 72)

Organism	Number (%)
*Staphylococcus* Sp	28 (39)
*Streptococcus* Sp	2 (3)
Gram negative Sp	4 (6)
Anaerobes	4 (6)
Mixed growth	7 (10)
No growth	25 (35)
No sample	2 (3)

### Recurrence

Twelve (17%) patients suffered a clinical recurrence of infection during the follow-up period (ten tibia, two femur). Recurrence occurred a median of 22-month post-treatment (IQR 39.9, range 4–65). In six cases the organism isolated was the same as that identified during the index procedure, five *Staphylococcus *Sp*.* and one *Streptococcus Sp.* In the other six cases, the organism isolated was different to that initially identified. These included two *Staphylococcus *Sp.; one *Pseudomonas*; one *Enterobacter*; one *Escherichia Coli*; and one polymicrobial infection (*Streptococcus *Sp + *Staphylococcus *Sp + *E coli)*. There was no difference in time to recurrence between those patients in whom the same organism was isolated, and those where the organism was different (*p* = 0.50). Interestingly, two of the recurrent cases (one *Pseudomonas* and one *Staphylococcus *Sp*.*) did not grow an organism at the index procedure.

As would be expected those patients with recurrence required more procedures (5.7 vs 2.3 procedures; *p* < 0.01) and more frequent admissions (3.9 vs 2.2 admissions; *p* < 0.01). Recurrence rates were lower, where local antibiotics (10% with vs 33% without; *p* = 0.01), and RIA (6% with vs 20% without; *p* = 0.04) were employed, whilst they were increased when soft tissue reconstruction was required (33% with vs 10% without; *p* = 0.01) (Table 2).

**Table 2:** Impact of patient demographics, initial injury characteristics and treatment modalities on recurrence

Variable	Recurrence	No recurrence	*p* value
CM host classification			
A	5	33	0.46
Bs	5	23	
Bl	2	4	
Initial ISS			
< 16	8	43	0.73
≥ 16	4	17	
Soft tissue injury			
Open	5	14	0.17
Closed	6	42	
Site			
Tibia	9	36	0.33
Femur	3	24	
Local antibiotics			
Utilised	5	46	0.01
Not utilised	3	14	
Soft tissue reconstruction			
Required	7	14	0.01
Not required	1	46	
RIA			
Utilised	2	29	0.04
Not utilised	6	31	

*CM* Cierny–Mader, *ISS* Injury Severity Score, *RIA* Reamer Irrigator Aspirator

### Amputation

Nine patients underwent amputation, seven trans-tibial and two trans-femoral. This included eight patients with PTOM of their tibia, and one with PTOM of the femur. In four cases an amputation was performed following failed attempts at limb salvage, including two with uncontrollable recurrent disease. The remaining five patients underwent an amputation having chosen this option following a discussion of limb salvage vs amputation, often in the context of significant ipsilateral complications of their original trauma (summarised in Table 3). Amputations were performed at a median of 47 months following diagnosis (IQR 81, range 8–174), following 5 operative procedures (IQR 2.5, range 0–7). One patient had recurrent disease within their tibial remnant which was successfully supressed with further debridement and local antibiotics. Trends were seen towards lower amputation rates when the disease affected the femur, and when the original injury was closed (Table 4).

**Table 3:** Reasons for amputation

Patient	PTOM site	Level of amputation	Reason for amputation	Complications
1	Tibia	Transtibial	Patient preference—ipsilateral failed ankle fusion with co-existing subtalar and midfoot arthritis	Scar revision
2	Tibia	Transfemoral	Patient preference—felt would not tolerate limb reconstruction techniques	Nil
3	Tibia	Transtibial	Patient preference—counselled limb recon vs amputation, chose amputation	Nil
4	Tibia	Transtibial	Patient preference—Multifocal femoral and tibial deformities, ipsilateral ankle + midfoot arthritis	Recurrence at 8 months, successfully treated with debridement + local antibiotics
5	Femur	Transfemoral	Patient preference—felt would not tolerate limb reconstruction techniques	Nil
6	Tibia	Transtibial	Failed limb salvage—failed masquelet	Nil
7	Tibia	Transtibial	Failed limb salvage—Recurrent disease	Scar revision
8	Tibia	Transtibial	Failed limb salvage—recurrent disease	Nil
9	Tibia	Transtibial	Failed limb salvage—failed distraction osteogenesis	Nil

**Table 4:** Impact of patient demographics, initial injury characteristics and treatment modalities on requirement for amputation

Variable	Amputation	No Amputation	*p* value
CM host classification			
A	4	34	0.27
Bs	3	25	
Bl	2	4	
Initial ISS			
< 16	6	47	0.26
≥ 16	3	16	
Soft tissue injury			
Open	4	15	0.07
Closed	3	45	
Site			
Tibia	8	38	0.09
Femur	1	25	
Recurrent disease			
Recurrence	2	10	0.63
No recurrence	3	53	
Local antibiotics			
Utilised	5	46	0.28
Not utilised	0	17	
Soft tissue reconstruction			
Required	3	13	0.47
Not required	2	45	
RIA			
Utilised	1	30	0.37
Not utilised	4	33	

### Patient reported outcome measures

Out of 72 patients, 18 died (25%) prior to final review. Another 10 patients were not available for review (4 moved out of area, 3 developed dementia and 3 were uncontactable). Overall, out of the remaining 44 patients, 37 patients (84%) agreed to participate and to provide PROMs. The demographics, BACH classification, microorganisms isolated, and surgical management of these patients are summarised in Tables 5, 6, and 7. When compared to the cohort of patients unavailable to provide PROMS, there were no differences in demographics, injury characteristics, or treatment strategy employed. PROMs were obtained at a median of 111 months (range 66–177 months) following diagnosis of PTOM.

**Table 5:** Demographics, initial injury classification and BACH classification of PROMs cohort (*n* = 37)

Variable	*n*
Median age, years (IQR, range)	47 (21, 18–72)
Gender, *n* (%)	
Male	33 (89)
Female	4 (11)
Site, *n* (%)	
Tibia	24 (65)
Femur	13 (35)
Soft tissue injury, *n* (%)	
Open fracture	12 (32)
Closed fracture	22 (60)
Unknown	3 (8)
Initial injury severity score	
< 16	25 (68)
> 16	12 (32)
Cierny–Mader host classification, *n* (%)	
A	25 (68)
Bs	7 (19)
Bl	5 (13)
BACH type, *n* (%)	
Uncomplicated	16 (43)
Complex	21 (57)
Bone involvement, *n* (%)	
B1	29 (78)
B2	5 (14)
B3	3 (8)
Antimicrobial options, *n* (%)	
Ax	11 (30)
A1	17 (46)
A2	9 (24)
Coverage of soft tissues, *n* (%)	
C1	27 (73)
C2	10 (27)
Host status, *n* (%)	
H1	31 (84)
H2	6 (16)

**Table 6:** Organisms isolated from PROMs cohort (*n* = 37)

Organism	Number (%)
Staphylococcus	17 (52)
Streptococcus	2 (6)
Anaerobes	3 (9)
Gram negative rod	1 (3)
Multi-organism	3 (9)
No growth	11 (30)

**Table 7:** Surgical management and follow-up of PROMs cohort (*n* = 37)

Variable	*N*
Debridement, *n* (%)	37 (100)
Removal of implants *n* (%)	31 (84)
Implants previously removed *n* (%)	4 (11)
No implants present *n* (%)	2 (5)
Local antibiotic provision *n* (%)	25 (68)
Spacer *n* (%)	10 (40)
Beads *n* (%)	4 (16)
Nail *n* (%)	3 (12)
Combination of above techniques *n* (%)	8 (32)
Soft tissue reconstruction, *n* (%)	10 (27)
Free flap *n* (%)	3 (30)
Local flap *n* (%)	1 (10)
Skin graft *n* (%)	1 (10)
Combination of above techniques *n* (%)	5 (50)
RIA, *n* (%)	15 (41)
Median number of procedures, *n* (IQR, range)	2 (3, 1–13)
Median duration of clinic follow-up, months (IQR, range)	23 (27, 6–119)

### Lower extremity functional scale

Within our cohort the median LEFS was 60 (IQR 40, range 21–80). Only two factors appeared to be associated with LEFS (Table 8): the utilisation of the RIA system (69.6 with vs 52.8 without; *p* = 0.02); and the overall BACH classification (74.4 for uncomplicated vs 58.4 for complex; *p* = 0.02). When examining the breakdown of the BACH classification, both the BACH coverage classification (66.4 for C1 vs 49.2 for C2; *p* = 0.01) and BACH host status (61.6 for H1 vs 42 for H2; *p* = 0.03) were associated with outcome.

**Table 8:** PROMs of patients undergoing treatment for PTOM. EQ-5D-3L presented as index score (*n* = 37)

Variable	Median LEFS	*p* value	Median EQ-5D-3L	*p* value	Median EQ-VAS	*p* value
CM host						
A	66.4	0.16	0.725	0.83	80	0.66
Bs	59.2		0.779		65	
Bl	58.4		0.815		70	
Site						
Tibia	58.8	0.41	0.735	0.66	75	0.52
Femur	66.4		0.760		80	
Soft tissue injury						
Open	76	0.07	0.805	0.10	85	0.17
Closed	60.4		0.708		67.5	
Local antibiotics						
Utilized	61.6	0.79	0.692	0.35	82.5	0.54
Not utilized	56.8		0.760		70	
RIA						
Utilized	69.6	0.03	0.883	0.02	80	0.14
Not utilized	52.8		0.604		62.5	
Amputation						
Required	46.8	0.18	0.718	0.72	72.5	0.69
Not required	61.6		0.760		80	
Recurrent disease						
Recurrence	70.4	0.62	0.796	0.85	87.5	0.65
No recurrence	60		0.725		70	
BACH type						
Uncomplicated	74.4	0.009	0.778	0.41	80	0.33
Complex	58.4		0.725		70	
Bone involvement						
B1	61.6	0.16	0.779	0.55	65	0.17
B2	66.4		0.725		80	
B3	30.4		0.189		30	
Antimicrobial options						
Ax	60	0.15	0.656	0.28	60	0.04
A1	72		0.833		85	
A2	48		0.587		60	
Coverage of the soft tissues						
C1	66.4		0.779		80	0.04
C2	49.2		0.516		65	
		0.01		0.05		
Host status						
H1	61.6		0.760		80	0.97
H2	42	0.03	0.435	0.47	80	

### EQ-5D-3L and EQ-VAS

The median EQ-5D-3L index score was 0.760 (IQR 0.484, range − 0.239 to 1.000), and the median EQ-VAS was 80 (IQR 40, range 12–100). The use of RIA was associated with significantly higher EQ-5D-3L scores (0.883 with vs 0.604 without; *p* = 0.04) (Table 8). This was not, however, seen with EQ-VAS (80 with vs 62.5 without; *p* = 0.14). Unlike LEFS, overall BACH classification did not demonstrate association with either EQ-5D-3L (0.778 for uncomplicated vs 0.725 complex), or EQ-VAS (80 for uncomplicated vs 70 for uncomplicated). We did, however, note significantly higher scores in the EQ-VAS when wider antimicrobial options were available (85 for A1 vs 60 for A2 and 60 for Ax; *p* = 0.04), and when soft tissue reconstruction was not required (80 for C1 vs 65 for C2; *p* = 0.04).

## Discussion

Within PTOM, prior literature reports a significant level of morbidity including impaired ambulation, chronic pain, and an inability to re-secure employment [30, 31]. These studies frequently focus on binary outcomes which do not reflect the functional deficit that many patients continue to live with, despite no active signs of residual infection. To our knowledge this study presents one of the largest cohort of limb specific PROMS in patients with lower limb PTOM, with the longest follow-up published to date. We demonstrate the ongoing morbidity suffered by this patient group, with levels of function below that of the average population, even following “cure”.

### Recurrence

Prior literature reports that 90% of recurrences occur within 2 years of treatment for post-traumatic osteomyelitis [32, 33]. It has been noted that patients presenting more than 3 years following treatment had usually sustained a new injury, undergone a further procedure, or isolated different organisms suggesting that they are re-infections as opposed to recurrence [33]. Within our cohort the rate of recurrence was 17%, with a median time to recurrence being 22 months. Only seven patients presented within 2 years (2-year recurrence rate 9.7%). None of our patients presenting after 2 years had sustained further injury or undergone further operative procedures. In six cases, the organism isolated at the revision procedure was different to that originally isolated; however, there was no difference in the time to presentation between those patients, where the recurrence organism was the same as the original, and those where it differed.

Previously recorded risk factors for recurrence include increasing age, symptoms for more than 3 months prior to treatment, pseudomonal infection, and non-operative treatment [34, 35]. Within our cohort we observed reduced recurrence rates with the use of RIA as an adjunct to debridement, and when soft tissue reconstruction was not required. RIA is used to debride and irrigate the canal in patients with medullary sepsis [35]. We observed a 6% recurrence rate in these patients compared with 20% in those receiving other treatments. This reduction may represent a selection bias towards patients with less complex bone lesions, as opposed to a treatment effect. Previous reports are consistent with low recurrence rates following treatment of medullary sepsis using this approach [36–38]. Similarly, we would also propose that patients requiring soft tissue reconstruction are likely to have more severe disease, potentially suggesting that disease pattern and severity are likely the most important determinants of recurrence.

Recurrence rates were also significantly lower when local antibiotic void fillers were utilised at the surgical site, a technique that is well-described. These provide very high concentrations of antibiotics whilst occupying dead space and preventing haematoma formation and suppuration. Several studies have previously reported significant improvement in outcome associated with the use of these agents compared with standard of care. Recurrence rates in case series vary from 0 to 24%.[39–42]. In our series the recurrence rate was 10%, where local antibiotics were used, we now use these agents as standard in keeping with the majority of current treatment algorithms [38].

### Amputation

Studies examining amputation in PTOM report rates ranging from 1% to 7%. Within our cohort, the amputation rate was 12.5%. Amputation is frequently utilised as a surrogate for poor outcome or treatment failure; however, there is an increasing body of literature supporting early amputation under circumstances, where the outcome is likely to be poor, or the morbidity of treatment high. Multiple studies in trauma have demonstrated that early amputation results in fewer overall complications, more rapid resolution of symptoms and equivalent or superior functional outcomes to those undergoing limb reconstruction techniques [44–47]. It is important, therefore, that the concept of functional amputation, where this approach is considered as a treatment choice rather than a last resort, is developed and discussed with patients at an early stage. Within our cohort, four amputations were performed because of failure of treatment in this cycle. In five cases amputation was performed as the primary treatment for PTOM at that point. Patients who underwent amputation reported equivalent EQ-5D-3L index scores (median 0.718 with vs 0.760) and EQ-VAS scores (median 72.5 with vs 80), suggesting that in complex cases, amputation can provide a successful and equivalent outcome. EQ-5D-3L and EQ-VAS were also satisfactory in the patient who had recurrence in their tibial remnant post-amputation (0.815 and 90, respectively).

### Patient reported outcome measures

The use of PROMS to record more meaningful outputs for research and monitoring of clinic care is increasing. Through improved understanding of the patient perspective, these can facilitate better communication, improved decision making, and greater patient satisfaction with care [40]. In patients with PTOM such measures may allow better recognition of the functional outcomes achieved by patients which can be used either to compare treatment strategies, or counsel patients prior to embarking with treatment.

Within our cohort the median LEFS was 60, substantially lower than those scores recorded for a healthy population (median score 77) [41]. They are similarly lower than those reported in patients suffering lower limb injury, with a LEFS of 69.4 reported at 24 weeks following ankle fractures. This suggests that outcomes in the PTOM population are substantially worse than those experienced within the standard fracture population [42]. The scores recorded by our patients were similar to those reported in two previous studies using the same measure in PTOM. The LEFS recorded in 12 patients treated for osteomyelitis of their tibia, despite a 100% infection remission rate at 50-month follow-up, was an median of 51 [31]. Similarly, another series including 35 cases of tibial or femoral osteomyelitis, with an infection remission rate of 97% at a mean of 29.5 months, reported a mean LEFS of 65.5 [43]. Neither of these studies identified demographic or operative factors associated with an improved LEFS. Within our cohort we noted that the use of RIA was associated with significantly improved outcomes, as was the classification of osteomyelitis as BACH complex.

Quality of life indicators in the form of the EQ-5D-3L index score and the EQ-VAS also demonstrated that patients failed to return to the functional baseline of the general population (0.617 vs 0.856 and 68.5 vs 82.8, respectively). Two studies have examined EQ-5D-3L and EQ-VAS in PTOM. In a series of 9 patients with polymicrobial osteomyelitis of the femur, a mean EQ-5D-3L index score of 0.360, and a mean EQ-VAS of 61.6 were reported at 21.4 months [44]. In this series all cases were classified as complex, potentially explaining the poorer outcomes achieved. Another study used the same measures to assess outcomes in 56 patients with tibial and femoral osteomyelitis, recording that both EQ-5D-3L index score and EQ-VAS improved from 0.284 and 58.2, respectively, to 0.740 and 78.9 at 1-year post-op, closely matching those scores seen within the general population [45]. When stratifying patients according to the BACH classification, significantly higher EQ-5D-3L and EQ-VAS scores were recorded in those with uncomplicated disease, simpler bone lesions, and limited or well-controlled co-morbidities [45]. The overall results reported in this study are higher than those achieved within our population; however, we do not have data to assess our cohorts’ functional level at baseline, and, therefore, cannot make comparisons with regard to the degree of improvement. In addition, the follow-up time in our patients is much longer at a median of 111 months, making any comparison difficult to interpret. The literature reporting PROMs in tibial and femoral PTOM is summarised in Table 9 and compared to the findings of the current study.

**Table 9:** Previous literature examining PROMs in patients with tibial/femoral PTOM, compared with the current study

Author	Year	Number of cases	Management	Follow-up, Mean (range)	Functional outcome
Egol et al. [46]	2009	Tibia: 24 Femur: 16	Debridement + saline irrigation	21 months (10–57)	SMFA: Mean dysfunction score 53.8 (range 41.9–76.3). Mean bother index 51.5 (range 42.6–73.9)
Campbell et al. [31]	2011	Tibia: 12	Debridement, local antibiotics, ex-fix + soft tissue coverage	50 months (26–72)	LEFS: Median score 51 (range 14–80)
Wu et al. [43]	2017	Tibia: 16 Femur: 19	Debridement, local antibiotics, fixation (internal—26, external 9) + soft tissue coverage (8 pts)	29.5 months (24–45)	LEFS: Mean score 65.6 (range 37–80)
Li et al. [47]	2019	Tibia: 18	Debridement, local antibiotics, ex-fix + soft tissue coverage	29.7 months (24–36)	Enneking score: pre-operatively: 9.78 ± 1.26, post-operatively: 24.44 ± 4.27 (representing 81.5% of normal function)
Hotchen et al. [45]	2020	Tibia: 34 Femur: 22	Debridement + local antibiotics ± fixation if indicated	12 months	EQ-5D-3L index score: pre-operatively: 0.284, 1 year post-operatively: 0.740 EQ-VAS: Pre-operatively: 58.2, 1 year post-operatively: 78.9
Arshad et al. [44]	2021	Femur: 14	Debridement + local antibiotics ± fixation if indicated	21.4 months (7–49)	EQ-5D-3L index: Mean score 0.360 EQ-VAS: Mean score 61.6
Current study	2021	Tibia: 24 Femur: 13	Debridement ± local antibiotics, ± fixation ± soft tissue coverage	111 months (66–177)	LEFS: Median score 60 (range 21–80) EQ-5D-3L: Median score 0.760 EQ-VAS: Median score 80

*LEFS* lower extremity functional scale, *SMFA* short musculoskeletal function assessment

### Limitations

This study has limitations which should be considered when interpreting its findings. The study is retrospective in nature, with a moderate sample size, common to similar studies, reflecting the nature of this clinical condition. The case types are heterogenous in nature and different distributions and severity of disease are treated by differing methods. The severity and nature of the underlying condition more likely effects outcome, therefore, than the methods employed, which will be subject to significant selection bias. From the original cohort only 51% were available for PROMS collection, and whilst there were no differences in the considered variables between these groups, there may well be other inherent differences biasing these results. We excluded non-operatively managed PTOM as well as infected non-unions, and therefore, the conclusions drawn from this study cannot be applied to these patient cohorts.

## Conclusions

With modern techniques, recurrence rates within the PTOM literature remain low. Amputation, whilst still undertaken in certain cases, is not associated with a significant reduction in quality of life, and does not necessarily represent a failure of treatment. PROMS demonstrate a quality of life that remains below that of the general population, though this appears to be improved in certain clinical situations. The data presented here on long-term outcomes should help counsel patients regarding likely functional recovery.

## References

[1] Mouzopoulos G, Kanakaris NK, Kontakis G, Obakponovwe O, Townsend R, Giannoudis PV (2011). Management of bone infections in adults: the surgeon’s and microbiologist’s perspectives. Injury.

[2] Sia IG, Berbari EF (2006). Infection and musculoskeletal conditions: osteomyelitis. Best Pract Res Clin Rheumatol.

[3] Singh J, Rambani R, Hashim Z, Raman R, Sharma HK (2012). The relationship between time to surgical debridement and incidence of infection in grade III open fractures. Strateg Trauma Limb Reconstr.

[4] Lima ALL, Oliveira PR, Carvalho VC, Cimerman S, Savio E (2014). Recommendations for the treatment of osteomyelitis. Braz J Infect Dis.

[5] Huh J, Stinner DJ, Burns TC, Hsu JR (2011). Infectious complications and soft tissue injury contribute to late amputation after severe lower extremity trauma. J Trauma.

[6] Roddy E, Patterson JT, Kandemir U (2020). Delay of antibiotic administration greater than 2 hours predicts surgical site infection in open fractures. Injury.

[7] Hull PD, Johnson SC, Stephen DJG, Kreder HJ, Jenkinson RJ (2014). Delayed debridement of severe open fractures is associated with a higher rate of deep infection. Bone Jt J..

[8] Joseph CM (2020). Time to debridement in open high-grade lower limb fractures and its effect on union and infections: a prospective study in a tropical setting. J Orthop Surg (Hong Kong).

[9] Leibovici L, Yehezkelli Y, Porter A, Regev A, Krauze I, Harell D (1996). Influence of diabetes mellitus and glycaemic control on the characteristics and outcome of common infections. Diabet Med.

[10] Calhoun JH, Cobos JA, Mader JT (1991). Does hyperbaric oxygen have a place in the treatment of osteomyelitis?. Orthop Clin North Am.

[11] Neale TJ, Muir JC, Mills H, Horne JG, Jones MR (1987). Candida albicans vertebral osteomyelitis in chronic renal failure. Postgrad Med J.

[12] Lew DP, Waldvogel FA (2004). Osteomyelitis. Lancet (Lond, Engl).

[13] Castillo RC, Bosse MJ, MacKenzie EJ, Patterson BM (2005). Impact of smoking on fracture healing and risk of complications in limb-threatening open tibia fractures. J Orthop Trauma.

[14] Mader JT, Ortiz M, Calhoun JH (1996). Update on the diagnosis and management of osteomyelitis. Clin Podiatr Med Surg.

[15] Ferguson J, Alexander M, Bruce S, O’Connell M, Beecroft S, McNally M (2021). A retrospective cohort study comparing clinical outcomes and healthcare resource utilisation in patients undergoing surgery for osteomyelitis in England: a case for reorganising orthopaedic infection services. J Bone Jt Infect.

[16] Simpson AH, Deakin M, Latham JM (2001). Chronic osteomyelitis. The effect of the extent of surgical resection on infection-free survival. J Bone Jt Surg Br.

[17] Kanakaris NK, Tosounidis TH, Giannoudis PV (2015). Surgical management of infected non-unions: an update. Injury.

[18] Rightmire E, Zurakowski D, Vrahas M (2008). Acute infections after fracture repair: management with hardware in place. Clin Orthop Relat Res.

[19] Trampuz A, Zimmerli W (2008). Diagnosis and treatment of implant-associated septic arthritis and osteomyelitis. Curr Infect Dis Rep.

[20] Patzakis MJ, Zalavras CG (2005). Chronic posttraumatic osteomyelitis and infected nonunion of the tibia: current management concepts. J Am Acad Orthop Surg.

[21] Uçkay I (2006). Recurrent osteomyelitis caused by infection with different bacterial strains without obvious source of reinfection. J Clin Microbiol.

[22] Metsemakers W-J, Smeets B, Nijs S, Hoekstra H (2017). Infection after fracture fixation of the tibia: analysis of healthcare utilization and related costs. Injury.

[23] Metsemakers WJ (2018). Fracture-related infection: a consensus on definition from an international expert group. Injury.

[24] Baker SP, O’Neill B, Haddon WJ, Long WB (1974). The injury severity score: a method for describing patients with multiple injuries and evaluating emergency care. J Trauma.

[25] Cierny G, Mader JT, Penninck JJ (2003). A clinical staging system for adult osteomyelitis. Clin Orthop Relat Res.

[26] Hotchen AJ, Dudareva M, Ferguson JY, Sendi P, McNally MA (2019). The BACH classification of long bone osteomyelitis. Bone Jt Res.

[27] Binkley JM, Stratford PW, Lott SA, Riddle DL (1999). The lower extremity functional scale (LEFS): scale development, measurement properties, and clinical application. Phys Ther.

[28] EuroQol—a new facility for the measurement of health-related quality of life. Health Policy, 16(3):199–208, 1990, 10.1016/0168-8510(90)90421-9 10.1016/0168-8510(90)90421-9 10109801

[29] Conterno LO, Turchi MD (2013). Antibiotics for treating chronic osteomyelitis in adults. Cochrane Database Syst Rev.

[30] Siegel HJ, Patzakis MJ, Holtom PD, Sherman R, Shepherd L (2000). Limb salvage for chronic tibial osteomyelitis: an outcomes study. J Trauma.

[31] Campbell R, Berry MG, Deva A, Harris IA (2011). Aggressive management of tibial osteomyelitis shows good functional outcomes. Eplasty.

[32] Tice AD, Hoaglund PA, Shoultz DA (2003). Risk factors and treatment outcomes in osteomyelitis. J Antimicrob Chemother.

[33] McNally M, Ferguson J, Dudareva M, Palmer A, Bose D, Stubbs D (2017). For how long should we review patients after treatment of chronic osteomyelitis? An analysis of recurrence patterns in 759 patients. Orthop Proc.

[34] Garcia-Del-Pozo E, Collazos J, Carton JA, Camporro D, Asensi V (2018). Factors predictive of relapse in adult bacterial osteomyelitis of long bones. BMC Infect Dis.

[35] Kanakaris NK, Morell D, Gudipati S, Britten S, Giannoudis PV (2011). Reaming irrigator aspirator system: early experience of its multipurpose use. Injury.

[36] Kanakaris N, Gudipati S, Tosounidis T, Harwood P, Britten S, Giannoudis PV (2014). The treatment of intramedullary osteomyelitis of the femur and tibia using the Reamer-Irrigator-Aspirator system and antibiotic cement rods. Bone Jt J..

[37] Reilly RM, Robertson T, O’Toole RV, Manson TT (2016). Are antibiotic nails effective in the treatment of infected tibial fractures?. Injury.

[38] Metsemakers W-J (2020). Evidence-based recommendations for local antimicrobial strategies and dead space management in fracture-related infection. J Orthop Trauma.

[39] Qureshi MK, Ghaffar A, Tak S, Khaled A (2020). Limb salvage versus amputation: a review of the current evidence. Cureus.

[40] Nelson EC, Eftimovska E, Lind C, Hager A, Wasson JH, Lindblad S (2015). Patient reported outcome measures in practice. BMJ.

[41] Dingemans SA (2017). Normative data for the lower extremity functional scale (LEFS). Acta Orthop.

[42] Lin C-WC, Moseley AM, Refshauge KM, Bundy AC (2009). The lower extremity functional scale has good clinimetric properties in people with ankle fracture. Phys Ther.

[43] Wu H (2017). Two stage management of Cierny-Mader type IV chronic osteomyelitis of the long bones. Injury.

[44] Arshad Z, Aslam A, Lau E, Thahir A, Krkovic M (2021). Management of Polymicrobial Cierny-Mader grade 3 and 4 chronic osteomyelitis of the femur. Cureus.

[45] Hotchen AJ, Dudareva M, Corrigan RA, Ferguson JY, McNally MA (2020). Can we predict outcome after treatment of long bone osteomyelitis?. Bone Jt J..

[46] Egol KA, Singh JR, Nwosu U (2009). Functional outcome in patients treated for chronic posttraumatic osteomyelitis. Bull NYU Hosp Jt Dis.

[47] Li J, Zhang H, Qi B, Pan Z (2019). Outcomes of vacuum sealing drainage treatment combined with skin flap transplantation and antibiotic bone cement on chronic tibia osteomyelitis: a case series study. Med Sci Monit Int Med J Exp Clin Res.

